# Draft Genome Sequence of *Pseudomonas syringae* pv. persicae NCPPB 2254

**DOI:** 10.1128/genomeA.00555-15

**Published:** 2015-06-04

**Authors:** Wenjun Zhao, Hongshan Jiang, Qian Tian, Jie Hu

**Affiliations:** Institute of Plant Quarantine Research, Chinese Academy of Inspection and Quarantine, Beijing, China

## Abstract

*Pseudomonas syringae* pv. persicae is a pathogen that causes bacterial decline of stone fruit. Here, we report the draft genome sequence for *P. syringae* pv. persicae, which was isolated from *Prunus persica*.

## GENOME ANNOUNCEMENT

*Pseudomonas syringae* is responsible for causing diseases in more than 180 plant species, including fruit trees, vegetable crops, and flowers (1). The *P. syringae* complex comprises plant pathogens in eight closely related *Pseudomonas* spp., including 64 pathovars in *P. syringae* (2). *P. syringae* pv. persicae, the pathogen causing bacterial decline of stone fruit, which was first noted almost simultaneously in France and New Zealand, is now under quarantine control in Europe and China (3, 4). Here, we briefly describe the genome sequencing of *P. syringae* pv. persicae strain NCPPB 2254.

Three DNA libraries were constructed from the extracted genomic DNA using a NEB E7370L DNA library preparation kit for Illumina, with average insert sizes of 330 bp, 460 bp, and 600 bp, respectively. The sequencing was performed on an Illumina MiSeq platform using a MiSeq reagent kit v3, 600 cycles. A total of 2,931,855 × 2 × 301 bp generated reads were subjected to the skewer (5) program (version 0.1.123) with a quality threshold of 9 and an error threshold of 0.25 for adapter trimming, and then *de novo* assembled using the ABySS program (6) (version 1.5.2) with a k-mer size of 128. Fragments <300 bp long were removed from the final assembly. Annotations were made via the RAST (7) and xBASE2 (8) servers. As declared from the xBASE2 server, protein-coding gene finding, tRNA gene scanning, and rRNA gene identification were performed using Glimmer (9), tRNAscan-SE (10), and RNAmmer (11), respectively.

The draft genome comprises 201 contigs, with a G+C content of 58.44%, an *N*_50_ length of 69,352 bp, an *N*_90_ length of 19,310 bp, and a total length of 6.34 Mb. As annotated by the RAST server, it contains 5,625 protein-coding sequences (CDSs) and 86 RNA-coding sequences, among which 65 tRNA sequences and 21 rRNA clusters were found.

### Nucleotide sequence accession numbers.

The genome sequence has been deposited at GenBank under the accession no. LAZV00000000. The version described in this paper is version LAZV01000000.

## References

[1] Prokic A, Gasic K, Ivanovic MM, Kuzmanovic N, Sevic M, Pulawska J, Obradovic A 2012 Detection and identification methods and new tests as developed and used in the framework of cost873 for bacteria pathogenic to stone fruits and nuts. J Plant Pathol 94:S1.117–S1 126. doi:10.4454/jpp.v94i1sup.016.

[2] Young JM 2010 Taxonomy of *Pseudomonas syringae*. J Plant Pathol 92:5–24.

[3] Young JM, Jones DS, Gillings M 1996 Relationships between populations of *Pseudomonas syringae* pv. persicae determined by restriction fragment analysis. Plant Pathol 45:350–357. doi:10.1046/j.1365-3059.1996.d01-117.x.

[4] O’Brien HE, Thakur S, Guttman DS 2011 Evolution of plant pathogenesis in *Pseudomonas syringae*: a genomics perspective. Annu Rev Phytopathol 49:269–289. doi:10.1146/annurev-phyto-072910-095242. 21568703

[5] Jiang H, Lei R, Ding SW, Zhu S 2014 Skewer: a fast and accurate adapter trimmer for next-generation sequencing paired-end reads. BMC Bioinformatics 15:182. doi:10.1186/1471-2105-15-182. 24925680 PMC4074385

[6] Simpson JT, Wong K, Jackman SD, Schein JE, Jones SJ, Birol I 2009 ABySS: a parallel assembler for short read sequence data. Genome Res 19:1117–1123. doi:10.1101/gr.089532.108. 19251739 PMC2694472

[7] Aziz RK, Bartels D, Best AA, DeJongh M, Disz T, Edwards RA, Formsma K, Gerdes S, Glass EM, Kubal M, Meyer F, Olsen GJ, Olson R, Osterman AL, Overbeek RA, McNeil LK, Paarmann D, Paczian T, Parrello B, Pusch GD, Reich C, Stevens R, Vassieva O, Vonstein V, Wilke A, Zagnitko O 2008 The RAST server: Rapid Annotations using Subsystems Technology. BMC Genomics 9:75. doi:10.1186/1471-2164-9-75. 18261238 PMC2265698

[8] Chaudhuri RR, Loman NJ, Snyder LA, Bailey CM, Stekel DJ, Pallen MJ 2008 xBASE2: a comprehensive resource for comparative bacterial genomics. Nucleic Acids Res 36:D543–D546. doi:10.1093/nar/gkm928. 17984072 PMC2238843

[9] Delcher AL, Bratke KA, Powers EC, Salzberg SL 2007 Identifying bacterial genes and endosymbiont DNA with Glimmer. Bioinformatics 23:673–679. doi:10.1093/bioinformatics/btm009. 17237039 PMC2387122

[10] Lowe TM, Eddy SR 1997 tRNAscan-SE: a program for improved detection of transfer RNA genes in genomic sequence. Nucleic Acids Res 25:955–964. doi:10.1093/nar/25.5.0955. 9023104 PMC146525

[11] Lagesen K, Hallin P, Rødland EA, Staerfeldt HH, Rognes T, Ussery DW 2007 RNAmmer: consistent and rapid annotation of ribosomal RNA genes. Nucleic Acids Res 35:3100–3108. doi:10.1093/nar/gkm160. 17452365 PMC1888812

